# Prior Creation of the Lateral Extra‐articular Tenodesis Tunnel to Minimize Tunnel Convergence During Combined Anterior Cruciate Ligament Reconstruction

**DOI:** 10.1002/atn2.70297

**Published:** 2026-10-09

**Authors:** Makoto Suruga, Takashi Kozu, Yoshiyuki Yahagi, Hyunho Lee, Kazuyoshi Nakanishi

**Affiliations:** ^1^ Department of Orthopaedic Surgery Nihon University Hospital Tokyo Japan; ^2^ Department of Orthopaedic Surgery Nihon University Itabashi Hospital Tokyo Japan

## Abstract

Lateral extra‐articular procedures are increasingly combined with anterior cruciate ligament (ACL) reconstruction in revision and high‐risk primary cases; however, femoral tunnel convergence between ACL and lateral extra‐articular tenodesis (LET) tunnels remains a cause of iatrogenic graft compromise. We describe a simple technique to minimize tunnel interference when performing LET according to the modified Lemaire technique with bone tunnel fixation. The key concept is to create the LET femoral tunnel first and leave the passing pin in situ while creating the ACL femoral tunnel under fluoroscopic guidance, allowing real‐time adjustment of the guide pin trajectory. After arthroscopic confirmation that the passing pin is not visible within the ACL tunnel, it is removed while retaining a shuttle suture for graft passage. ACL reconstruction is completed in the standard manner, followed by LET graft preparation, passage posterior to the lateral collateral ligament, and fixation with a SwiveLock anchor with the lower leg in internal rotation. This sequence requires no specialized instruments and can be applied to other lateral extra‐articular procedures, including anterolateral ligament reconstruction. This technique provides a practical option to reduce femoral tunnel interference and protect the ACL graft during combined procedures.

## Graphical abstract

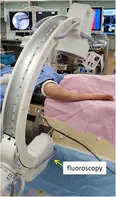

**VIDEO 1:** This video shows a surgical technique to prevent femoral tunnel convergence during anterior cruciate ligament (ACL) reconstruction combined with lateral extra‐articular tenodesis (LET) using the modified Lemaire technique. The patient is a 20‐year‐old man with an isolated right ACL injury and no concomitant meniscal pathology. The patient is positioned supine with the right knee flexed. A hamstring tendon autograft is used for ACL reconstruction, and the ACL femoral tunnel is created using the transportal technique through a far‐medial portal.The key concept of this technique is to create the LET femoral tunnel prior to the ACL femoral tunnel. The LET femoral tunnel is positioned just proximal and posterior to the femoral attachment of the lateral collateral ligament, and the passing pin is left in place during ACL tunnel drilling to serve as a real‐time reference for avoiding tunnel convergence under fluoroscopic guidance. A 1‐cm‐wide strip of the iliotibial band is harvested and passed posterior to the lateral collateral ligament using a looped suture, creating a pulley effect that enhances rotational control. The LET procedure is performed with the operative leg positioned off the edge of the operating table and supported on the surgeon's knee. After ACL reconstruction, the LET graft is fixed with a SwiveLock anchor with the knee in slight flexion and the lower leg held in internal rotation to ensure appropriate graft tension while avoiding overconstraint. This technique requires no specialized instruments and provides a simple and reproducible method to minimize the risk of graft damage caused by femoral tunnel convergence during combined ACL reconstruction with LET. Video content can be viewed at https://doi.org/10.1002/atn2.70297.

In 2013, Claes et al.^1^ provided a detailed anatomical description of the anterolateral ligament (ALL), which markedly increased interest in the anterolateral structures and their role in knee stability. This report renewed awareness of rotational instability associated with anterior cruciate ligament (ACL) injuries and led to rapid adoption of treatment strategies combining ACL reconstruction with lateral extra‐articular procedures (LEAP). Currently, LEAP is widely used as an augmentation technique, particularly in revision ACL reconstruction and in patients at high risk of graft failure.^2^, ^3^, ^4^, ^5^, ^6^, ^7^, ^8^

Lateral extra‐articular tenodesis (LET), one of the most representative LEAP techniques, has been used to control knee instability after ACL injury since before the establishment of intra‐articular ACL reconstruction.^9^, ^10^ Although ACL reconstruction is generally effective in restoring anterior stability, a subset of patients continues to experience residual rotational instability postoperatively.^11^, ^12^, ^13^ In this context, combining LET with ACL reconstruction has gained renewed attention.^2^, ^3^, ^14^

Biomechanical studies have shown that LET reduces the load applied to the ACL graft and limits anterior tibial translation during pivoting movements.^15^ Clinical studies have shown that LET significantly reduces rotational instability and lowers graft failure rates.^14^, ^16^, ^17^, ^18^, ^19^, ^20^, ^21^ Large database analyses have shown that LET utilization increased approximately 5‐fold between 2015 and 2023. Furthermore, an international consensus survey reported that the modified Lemaire technique was employed in 45.2% of LEAP procedures, making it the most commonly used extra‐articular augmentation technique worldwide.^2^, ^3^, ^4^, ^7^

At our institution, modified Lemaire LET is routinely performed as the first‐line augmentation technique.^9^ Although ALL reconstruction is regarded as anatomical,^17^, ^22^, ^23^ it requires additional graft harvesting and tibial fixation, resulting in greater surgical invasiveness.^24^, ^25^, ^26^ Therefore, LET is preferred as the primary augmentation procedure.

One potential complication associated with LET and ALL reconstruction is ACL graft damage caused by femoral tunnel convergence.^27^, ^28^, ^29^, ^30^ At our institution, bone tunnel fixation is preferred because it provides strong fixation and allows intraoperative adjustment of graft tension.^31^, ^32^ The purpose of this study is to describe a surgical technique to avoid ACL graft damage caused by femoral tunnel interference during LET. The indications for LET are summarized in Table 1.

**TABLE 1:** Indications for LET at Our Institution

**Category**	**Indications**
Revision ACL reconstruction	All revision cases
Primary ACL reconstruction—absolute indications	• All revision cases
	• Knee hyperextension >10°
	• Fixed anterior tibial subluxation
Primary ACL reconstruction—relative indications	• Grade 3 pivot‐shift test
	• Posterior tibial slope ≥12°
	• Generalized joint laxity
	• History of contralateral ACL injury
	• Patient age <20 yr

ACL, anterior cruciate ligament; LET, lateral extra‐articular tenodesis; yr, years.

## SURGICAL TECHNIQUES

ACL reconstruction is primarily performed using the transportal technique with the hamstring tendon autograft as the first‐line graft choice; however, the outside‐in technique is employed in select cases. LET is performed according to the modified Lemaire technique using a 1‐cm‐wide strip of the iliotibial band (ITB) passed posterior to the lateral collateral ligament (LCL). The surgical sequence is summarized in Table 2.

**TABLE 2:** Surgical Sequence

**Step**	**Procedure**
1	Graft harvesting
2	Creation of the LET femoral tunnel
3	Diagnostic arthroscopy and creation of the ACL femoral tunnel
4	Meniscal treatment when indicated and creation of the tibial tunnel
5	Fixation of the ACL graft
6	Harvesting and fixation of the LET graft

ACL, anterior cruciate ligament; LET, lateral extra‐articular tenodesis.

### Preoperative Setup

Prior to surgery, fluoroscopy is prepared and adjusted to allow imaging in the figure‐4 position (hip abduction and external rotation with knee flexion). The fluoroscopic unit is introduced from the ipsilateral side and adjusted to obtain a true lateral view of the femur (Figure 1). The key principle is to create the LET femoral tunnel before the ACL femoral tunnel using fluoroscopy during ACL femoral tunnel creation to avoid tunnel interference.

**FIGURE 1:**
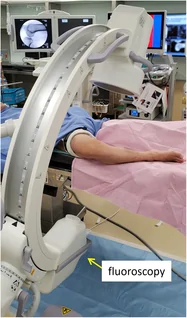
Intraoperative photograph showing the preoperative setup for the right knee. The patient is in the supine position with the knee positioned in the figure‐4 position (hip abduction, external rotation, and knee flexion). The fluoroscopic unit is introduced from the contralateral side and adjusted to obtain a true lateral view of the femur.

### LET Femoral Tunnel Preparation

A 3‐ to 5‐cm skin incision is made parallel to the ITB, centered midway between the lateral femoral epicondyle and the fibular head (Figure 2). The posterior border of the ITB is identified, and a 1‐cm‐wide strip is marked beginning 1 cm anterior to the posterior margin (Figure 3). At this stage, only the incision is made; graft release and suture placement are performed immediately before fixation (Video 1).

**FIGURE 2:**
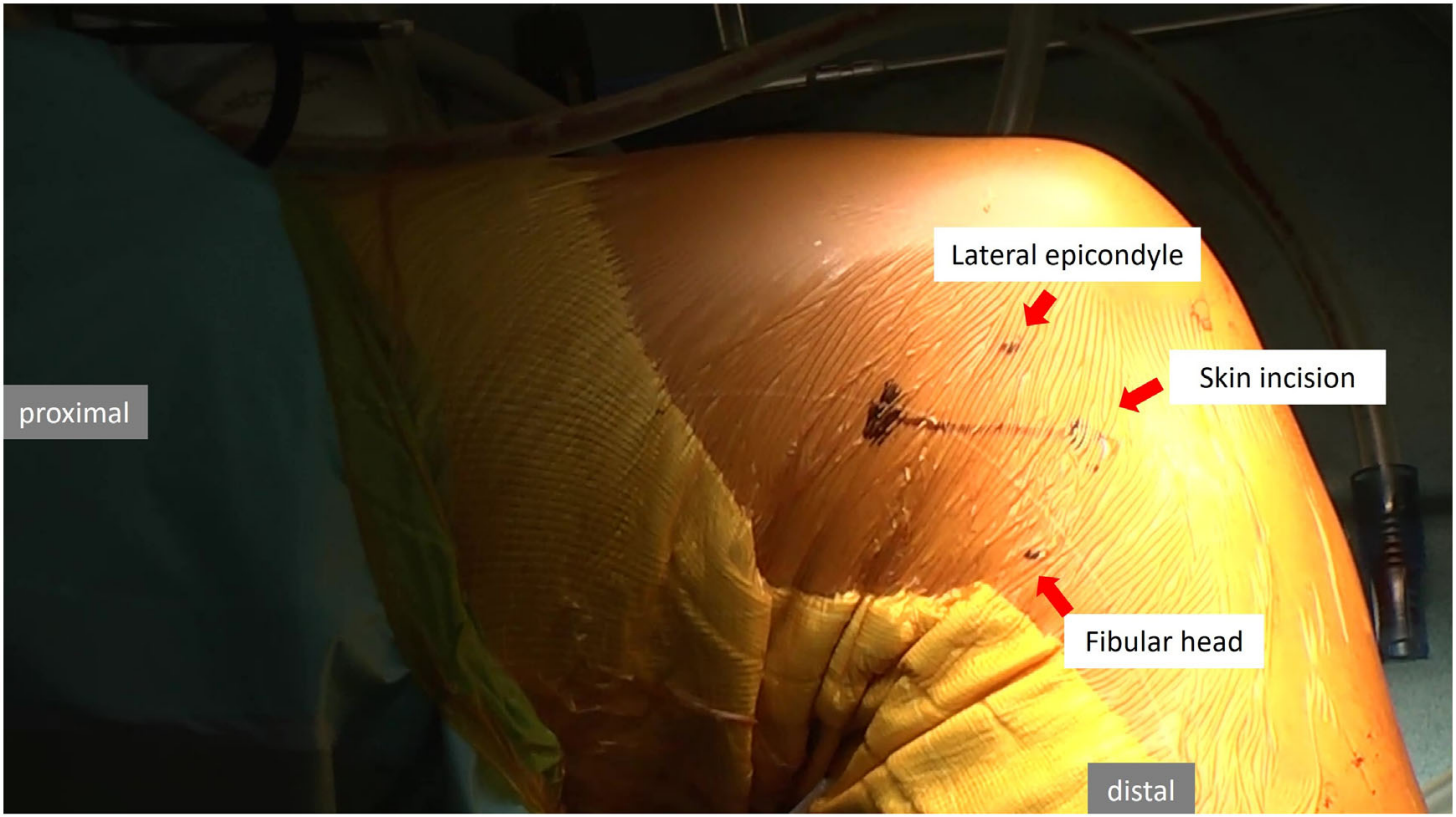
Intraoperative image with the patient supine and the right knee flexed to approximately 90°. A 3‐ to 5‐cm skin incision for LET is made parallel to the ITB, centered midway between the fibular head and the lateral femoral epicondyle. This incision provides access for both LCL identification and LET femoral tunnel creation. (ITB, iliotibial band; LCL, lateral collateral ligament; LET, lateral extra‐articular tenodesis.)

**FIGURE 3:**
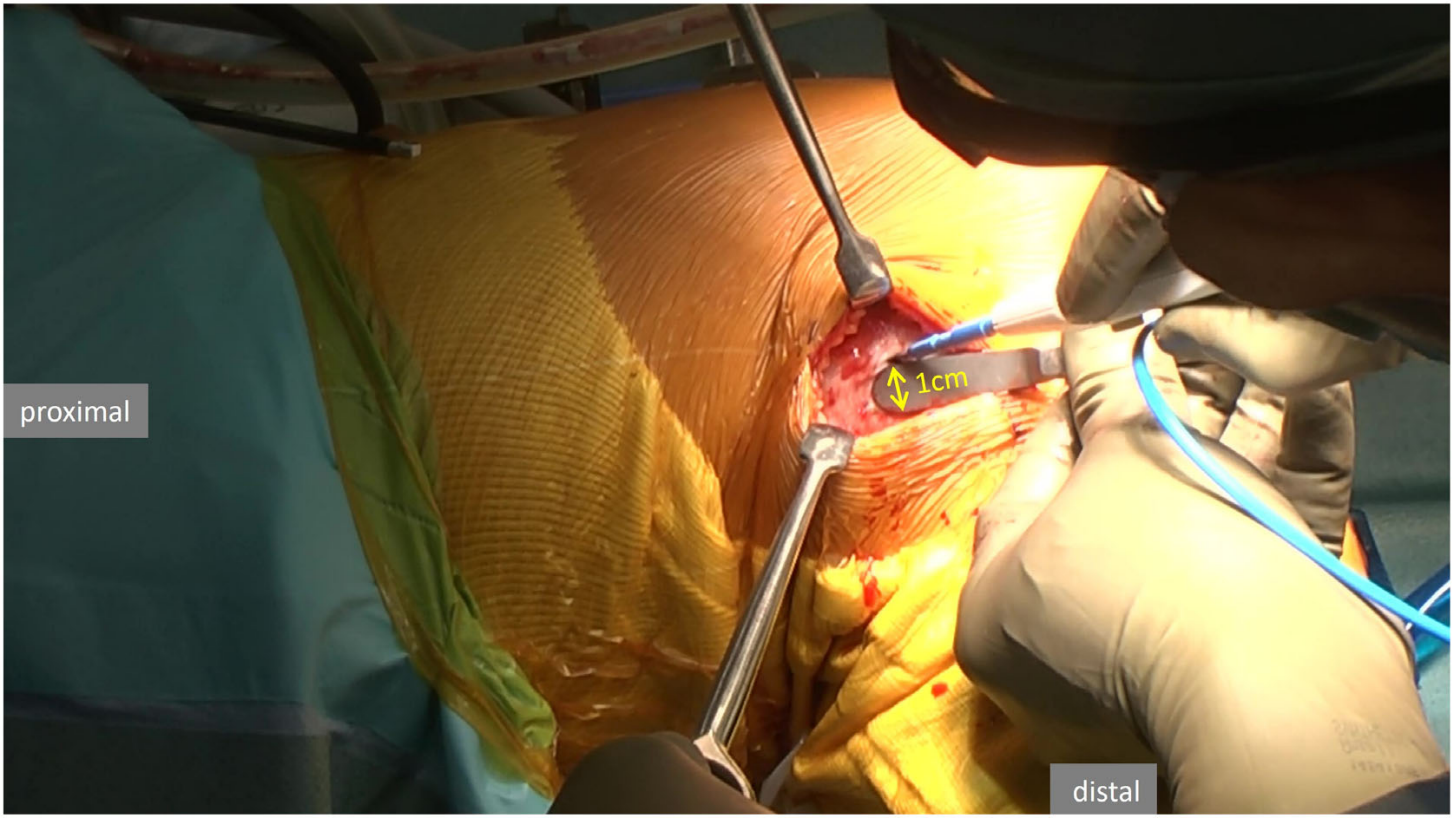
Intraoperative image with the patient supine and the right knee flexed to approximately 90°. The posterior border of the ITB is identified by palpation, and a 1‐cm‐wide strip is split starting 1 cm anterior to the posterior margin. The split extends distally to Gerdy's tubercle and proximally approximately 2 cm above the lateral femoral epicondyle. Graft release and suturing are performed later. (ITB, iliotibial band.)

The LCL is identified, and the surrounding soft tissues are mobilized (Figure 4). A looped suture is passed posterior to the LCL to avoid injury to the joint capsule. This looped suture is later used to pass the ITB graft posterior to the LCL.

**FIGURE 4:**
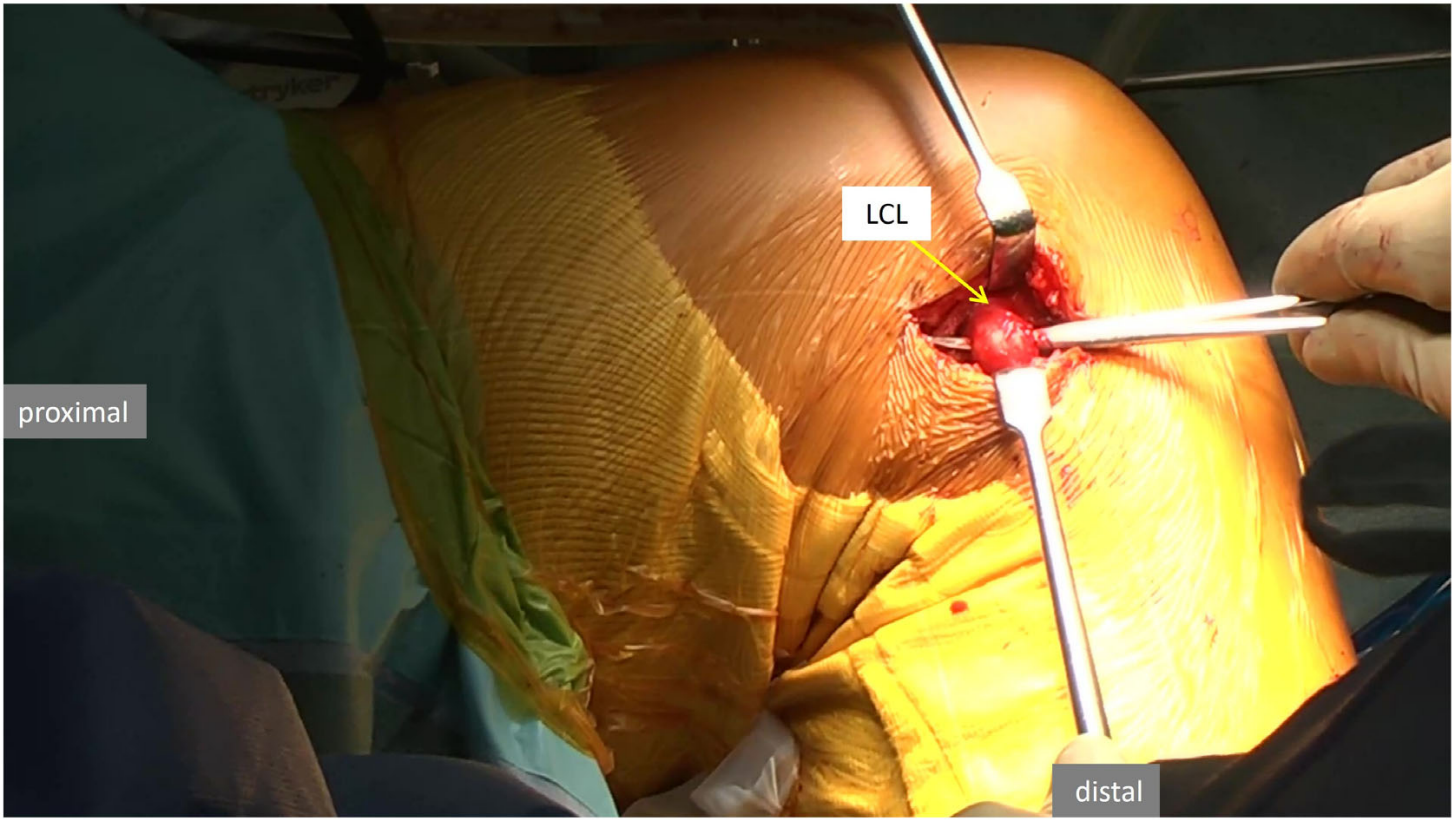
Intraoperative image with the patient supine and the right knee flexed to approximately 90°. Through the split ITB, the soft tissues are mobilized to identify the LCL. A looped suture is passed posterior to the LCL with careful dissection to avoid injury to the joint capsule. This suture is later used to pass the graft posterior to the LCL. (ITB, iliotibial band; LCL, lateral collateral ligament.)

By following the posterior fibers of the LCL, the posterior aspect of its femoral attachment is identified. A passing pin is inserted just proximal and posterior to the femoral attachment of the LCL, oriented perpendicular to the femoral shaft in the coronal plane and directed slightly anteriorly in the sagittal plane to minimize the tunnel convergence risk (Figure 5). Fluoroscopy confirms the appropriate insertion point and trajectory (Figure 6A and B). The LET femoral tunnel is then drilled over the passing pin (Figure 7), which is left in place while proceeding to ACL femoral tunnel creation (Figure 8).

**FIGURE 5:**
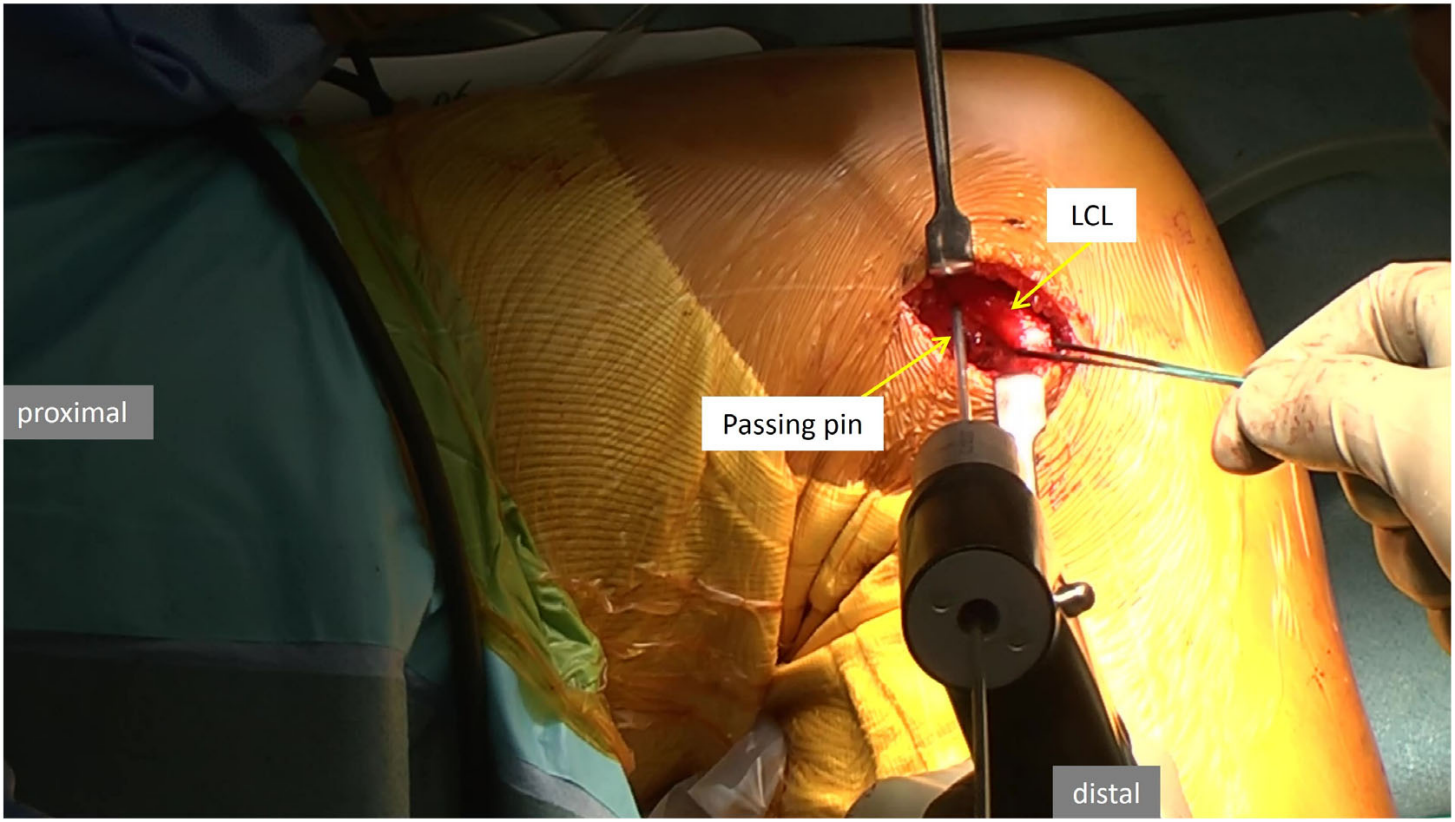
Intraoperative image with the patient supine and the right knee flexed to approximately 90°. After identifying the femoral attachment of the LCL, a passing pin is inserted just proximal and posterior to the attachment site. To prevent tunnel convergence, the pin is directed perpendicular to the femoral shaft, with a trajectory aimed to exit anterior to the medial femoral epicondyle. (LCL, lateral collateral ligament.)

**FIGURE 6:**
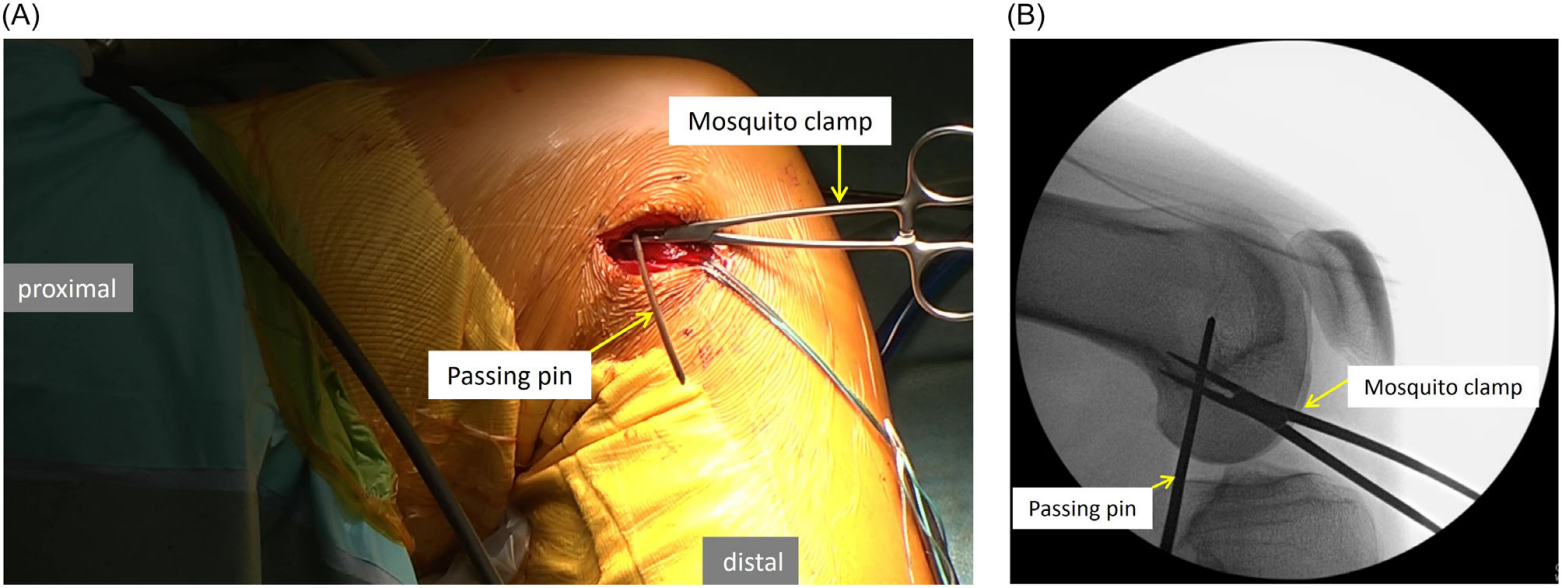
Intraoperative image with the patient supine and the right knee flexed to approximately 90°, showing fluoroscopic confirmation of tunnel position. (A) A mosquito clamp is attached to the passing pin insertion site as a radiographic marker. (B) The position and trajectory of the passing pin are confirmed under fluoroscopy.

**FIGURE 7:**
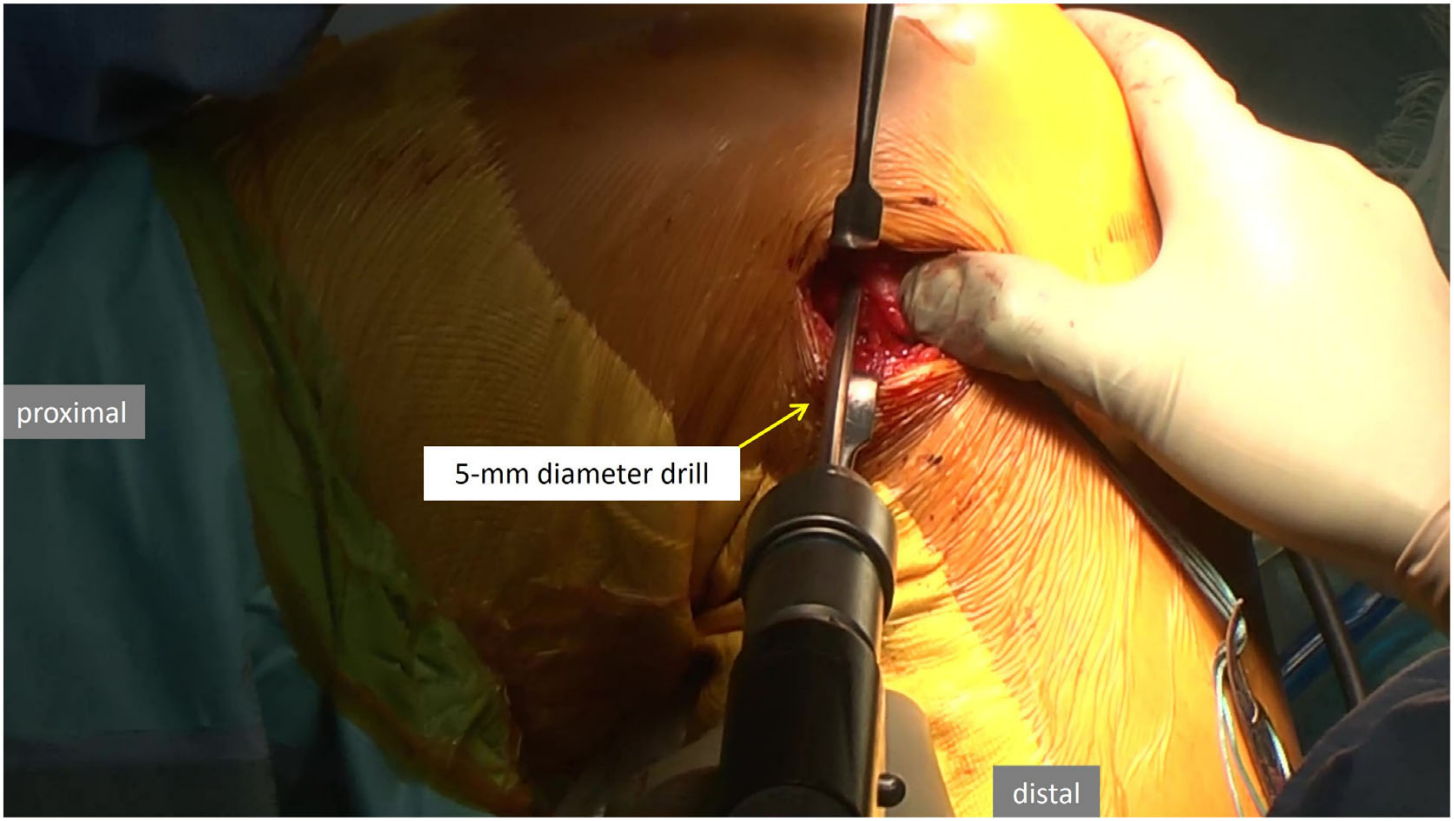
Intraoperative image with the patient supine and the right knee flexed to approximately 90°. A 5‐mm‐diameter drill is used to create the LET femoral tunnel to a depth of approximately 2 cm. This tunnel diameter accommodates the prepared ITB graft, and the depth allows adequate graft insertion for secure fixation. (ITB, iliotibial band; LET, lateral extra‐articular tenodesis.)

**FIGURE 8:**
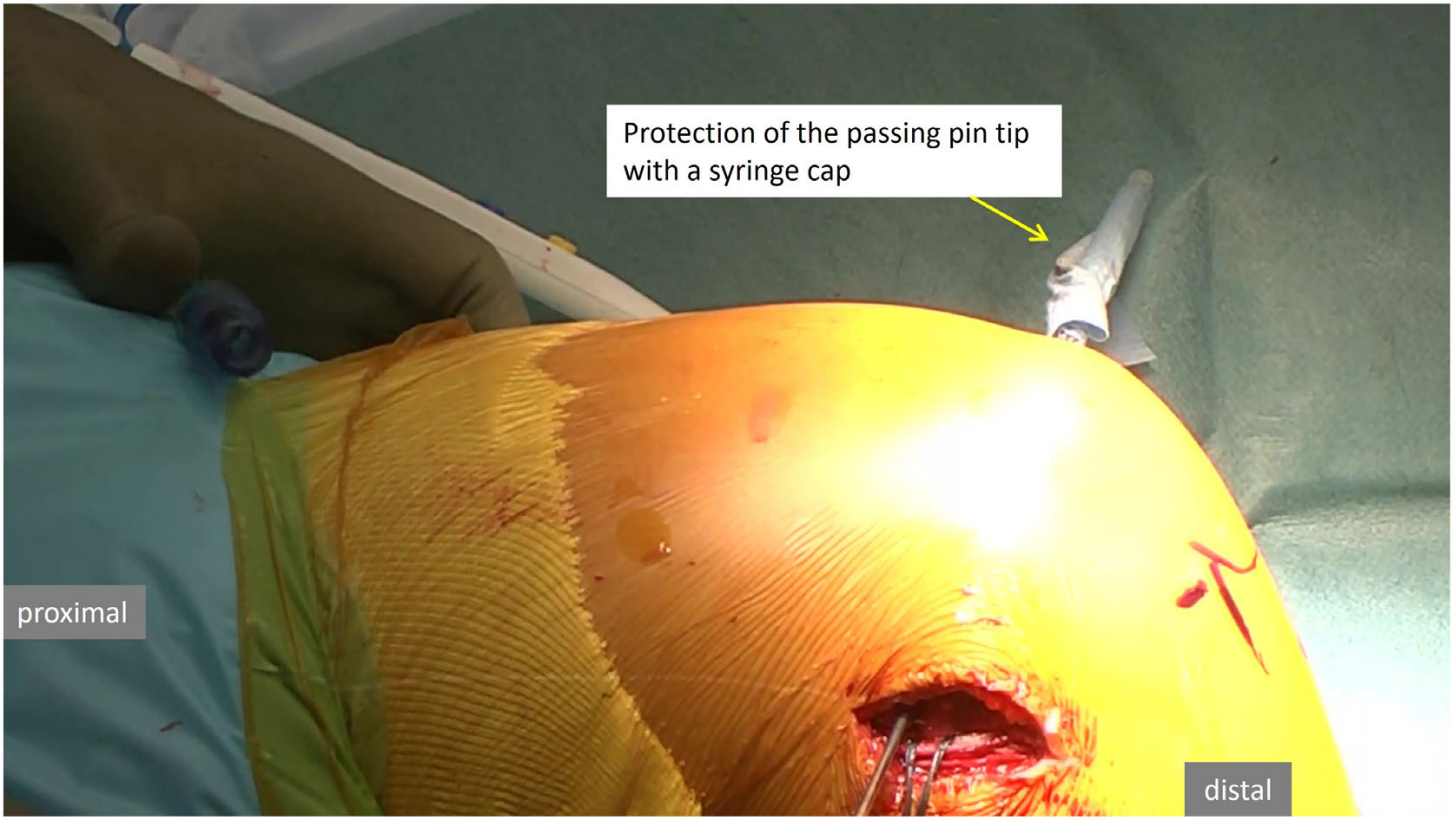
Intraoperative image with the patient supine and the right knee flexed to approximately 90°. After drilling, the passing pin is left in place to serve as a real‐time reference for avoiding tunnel convergence during ACL femoral tunnel creation. The pin tip is protected with a syringe cap secured with tape to prevent inadvertent injury. (ACL, anterior cruciate ligament.)

### Creation of the ACL Femoral Tunnel

The ACL femoral tunnel is created using the transportal technique with the knee in the figure‐4 position under fluoroscopic guidance. A guide pin is inserted while adjusting the trajectory to avoid interference with the LET passing pin (Figure 9A and B). If potential tunnel convergence is anticipated, the knee is flexed maximally and the guide pin trajectory is directed more proximally to avoid convergence. The exit point can be directly palpated through the lateral incision.

**FIGURE 9:**
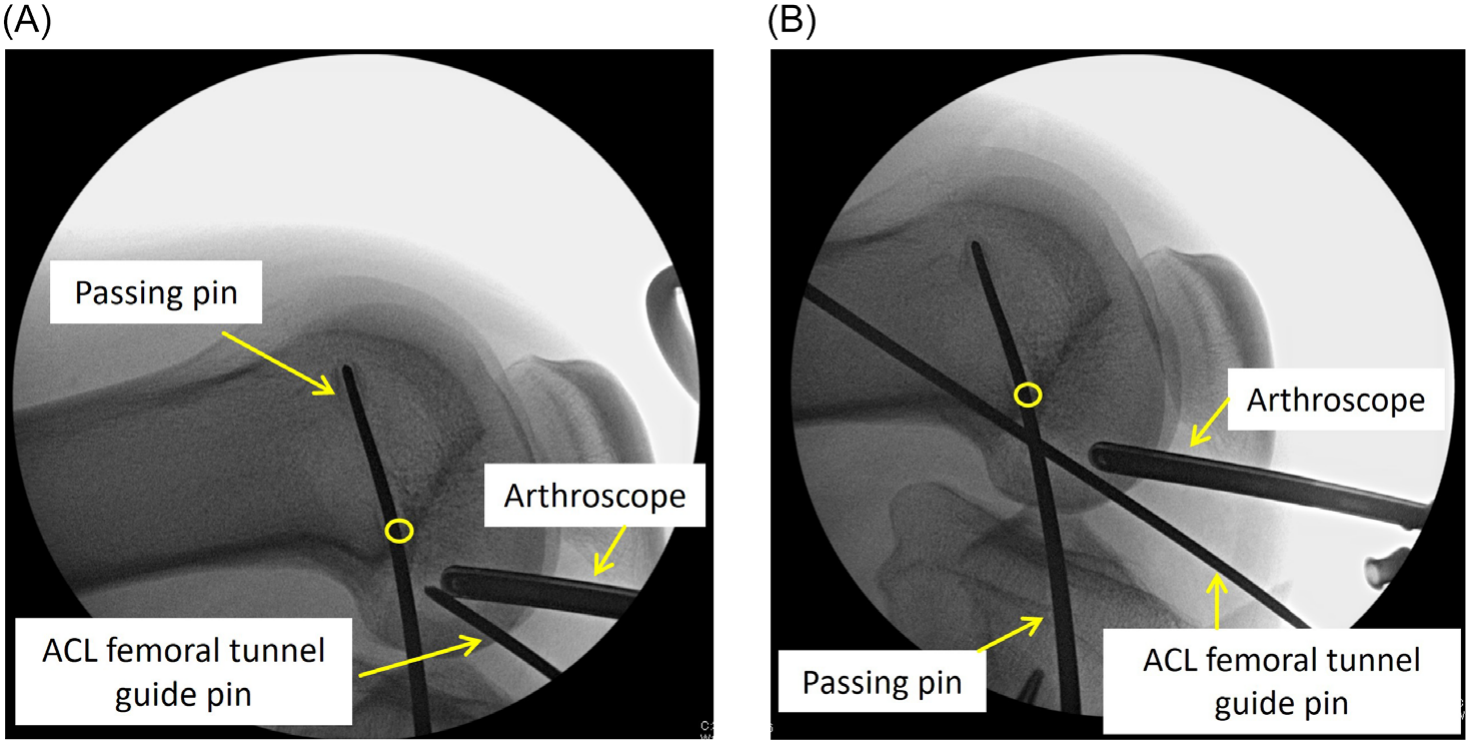
Intraoperative fluoroscopic image with the right knee in the figure‐4 position at maximal flexion. The yellow circle indicates the LET passing pin insertion site. (A) The position and trajectory of the ACL femoral tunnel guide pin are assessed using simultaneous fluoroscopic and arthroscopic visualization. (B) After guide pin insertion, no collision with the passing pin is observed, and tunnel convergence is not anticipated. (ACL, anterior cruciate ligament; LET, lateral extra‐articular tenodesis.)

After drilling, arthroscopic inspection confirms that the passing pin is not visible within the tunnel (Figure 10). Minor contact between tunnels is considered acceptable.^28^ A shuttle suture is tied to the passing pin, which is then removed while ensuring that the shuttle suture is securely retained (Figure 11). The outside‐in technique may offer greater flexibility in avoiding tunnel convergence.

**FIGURE 10:**
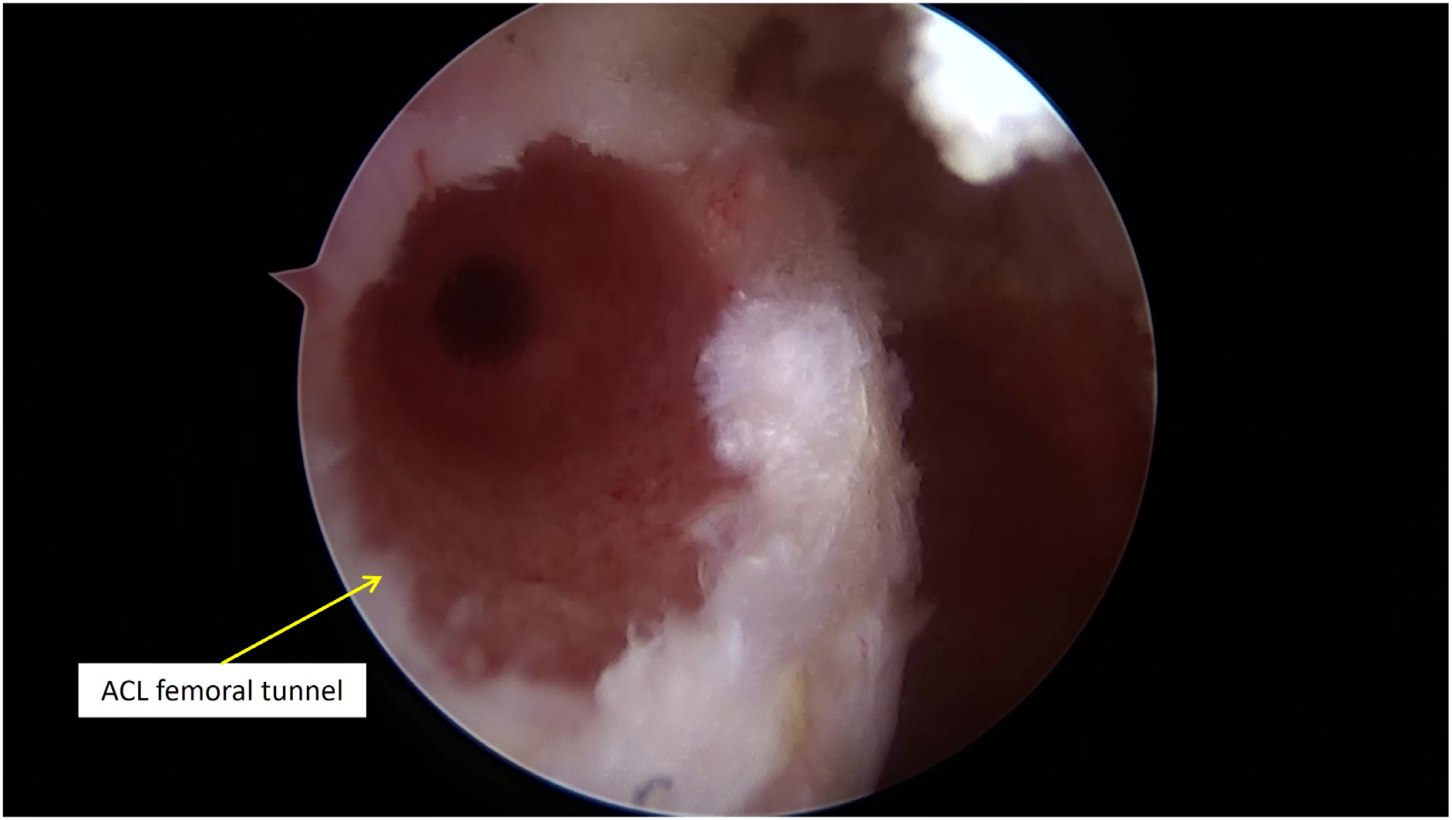
Arthroscopic image viewed from the anteromedial portal showing the ACL femoral tunnel after drilling in the right knee. The LET passing pin is not visible within the tunnel, confirming the absence of significant tunnel convergence and indicating that the ACL graft will not be compromised. (ACL, anterior cruciate ligament; LET, lateral extra‐articular tenodesis.)

**FIGURE 11:**
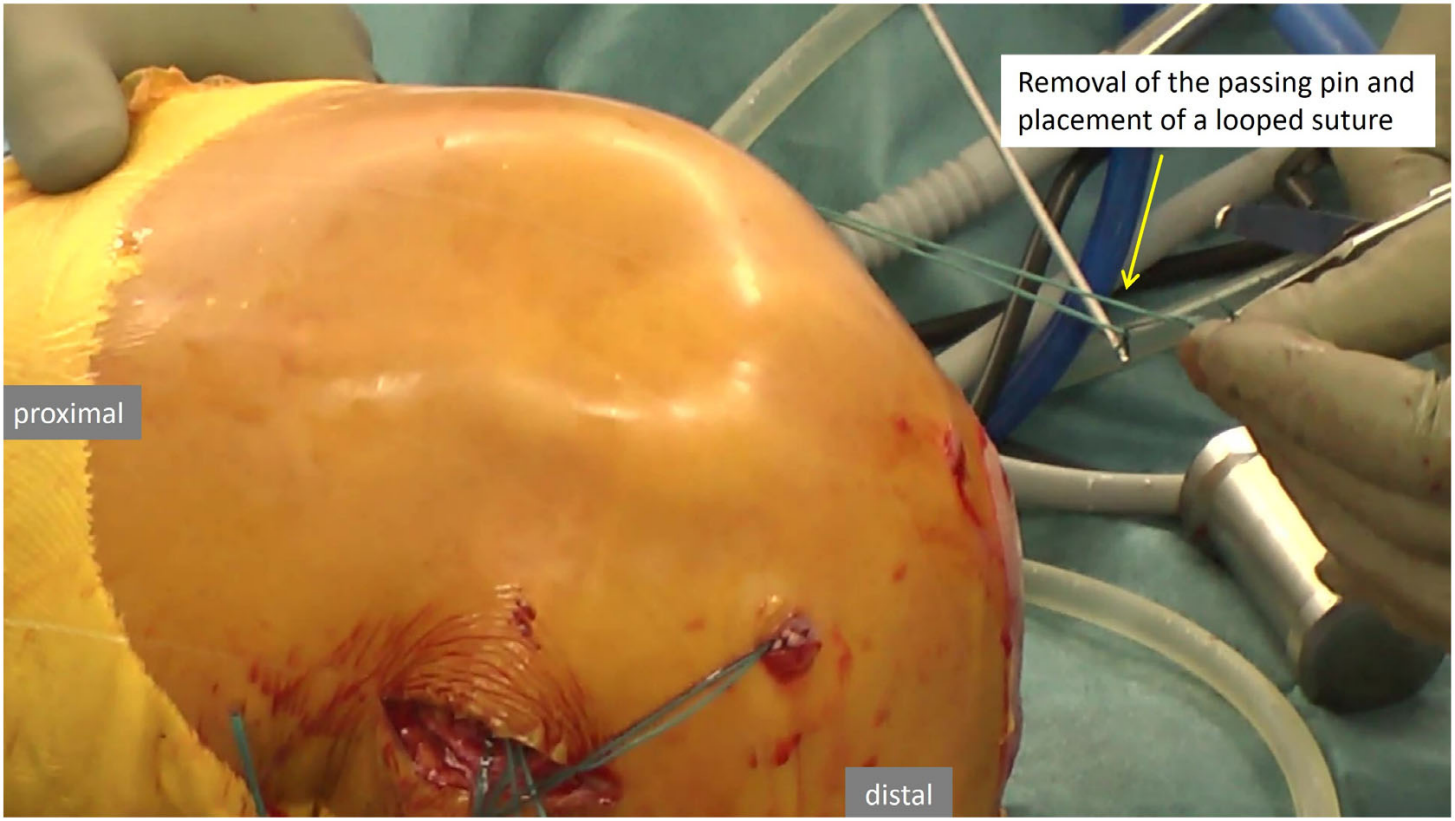
Intraoperative image with the patient supine and the right knee flexed to approximately 90°. After completion of the ACL femoral tunnel, a shuttle suture is tied to the passing pin, which is then removed. The shuttle suture is retained within the LET femoral tunnel to facilitate subsequent graft passage into the tunnel. (ACL, anterior cruciate ligament; LET, lateral extra‐articular tenodesis.)

### ACL Reconstruction, Meniscal Treatment, and LET Graft Fixation

Meniscal repair or partial meniscectomy is performed when indicated. The tibial tunnel is created, and the ACL graft is fixed first. Femoral fixation is achieved using an UltraButton (Smith & Nephew, Andover, MA), whereas tibial fixation is performed using a double‐spike plate (Smith & Nephew) with the knee in full extension.

LET graft fixation is performed as the final step. The graft is sutured using a Krackow stitch with SutureTape (Arthrex, Naples, FL) and adjusted to fit a 5‐mm tunnel (Figure 12). The prepared graft is passed posterior to the LCL using the looped suture (Figure 13) and guided into the femoral tunnel using the shuttle suture (Figure 14). With maximum tension applied from the contralateral side, an assistant maintains the lower leg in internal rotation, and fixation is performed using a SwiveLock 4.75‐mm anchor (Arthrex) (Figure 15A and B). Finally, the ITB is repaired using absorbable sutures. Postoperatively, computed tomography is performed to confirm appropriate tunnel positioning and verify the absence of significant tunnel convergence (Figure 16A and B). The postoperative rehabilitation protocol is summarized in Table 3, whereas Table 4 outlines the advantages and disadvantages of this technique, and Table 5 presents the pearls and pitfalls applicable to the procedure.

**FIGURE 12:**
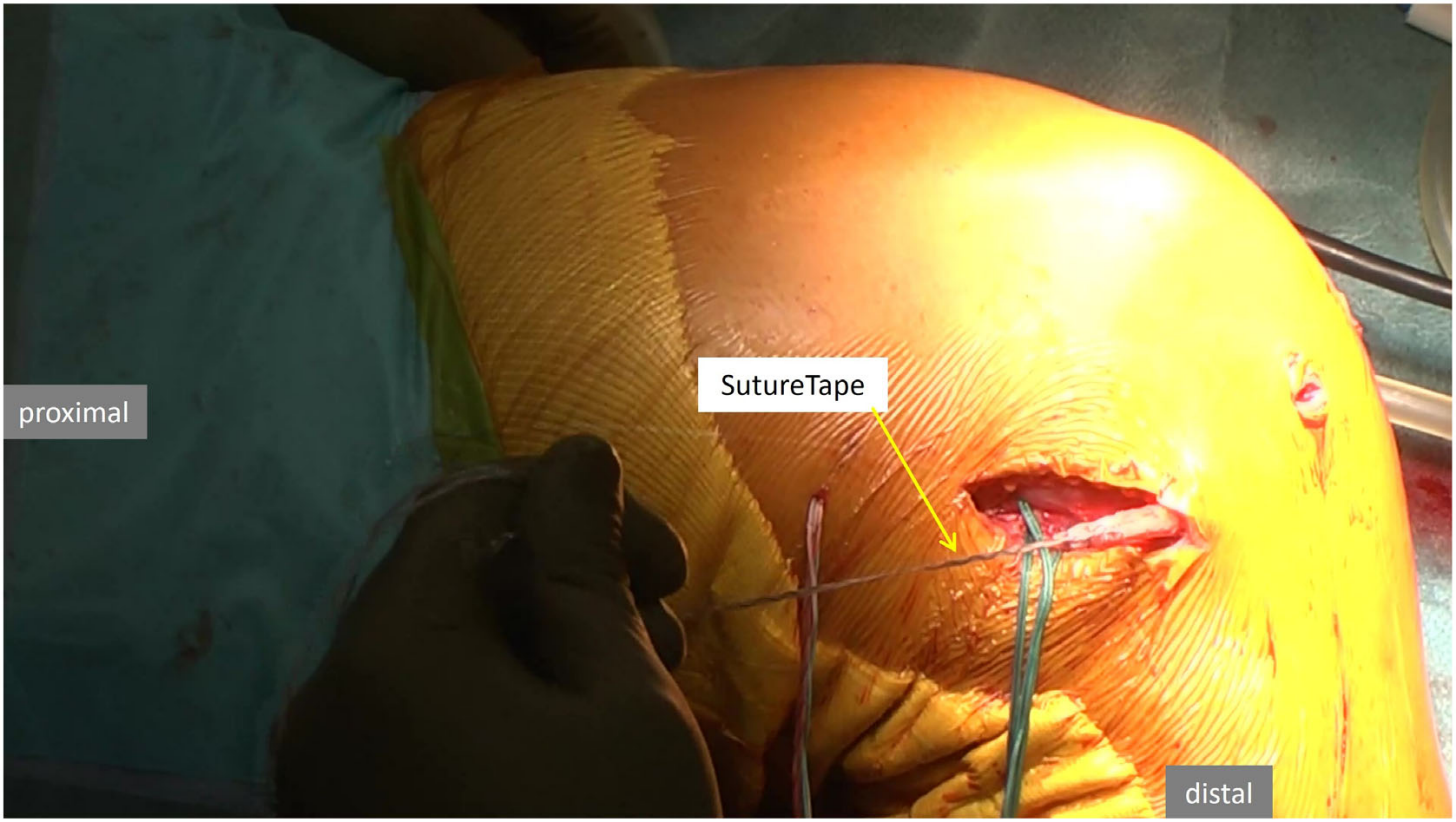
Intraoperative image with the patient supine and the right knee flexed to approximately 90°. The split ITB is transected to create a graft allowing approximately 1.5 cm of insertion into the femoral tunnel. After graft mobilization, a Krackow stitch is placed using SutureTape (Arthrex, Naples, FL). The graft diameter is confirmed to fit a 5‐mm tunnel. (ITB, iliotibial band.)

**FIGURE 13:**
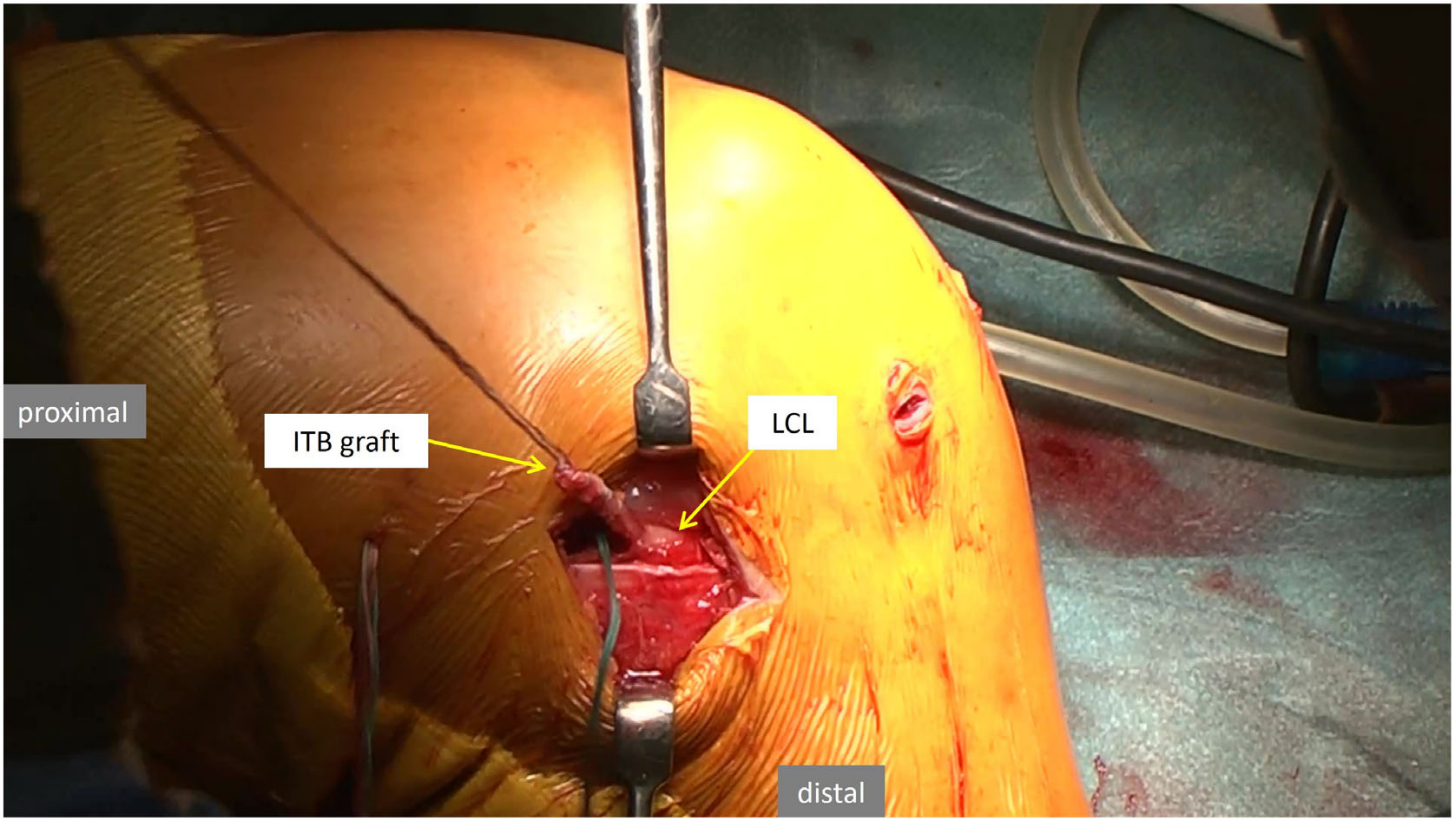
Intraoperative image with the patient supine and the right knee flexed to approximately 90°. The graft is passed posterior to the LCL using the previously placed looped suture. Passing the graft posterior to the LCL creates a pulley effect that enhances rotational control. (ITB, iliotibial band; LCL, lateral collateral ligament.)

**FIGURE 14:**
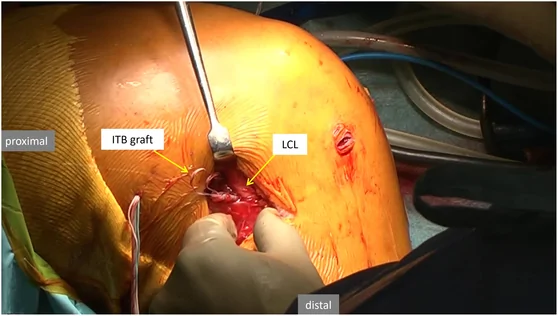
Intraoperative image with the patient supine and the right knee flexed to approximately 90°. Using the shuttle suture retained within the LET femoral tunnel, the graft is guided into the tunnel. Care is taken to confirm that the graft tip is fully seated within the tunnel to ensure adequate fixation strength. (ITB, iliotibial band; LCL, lateral collateral ligament; LET, lateral extra‐articular tenodesis.)

**FIGURE 15:**
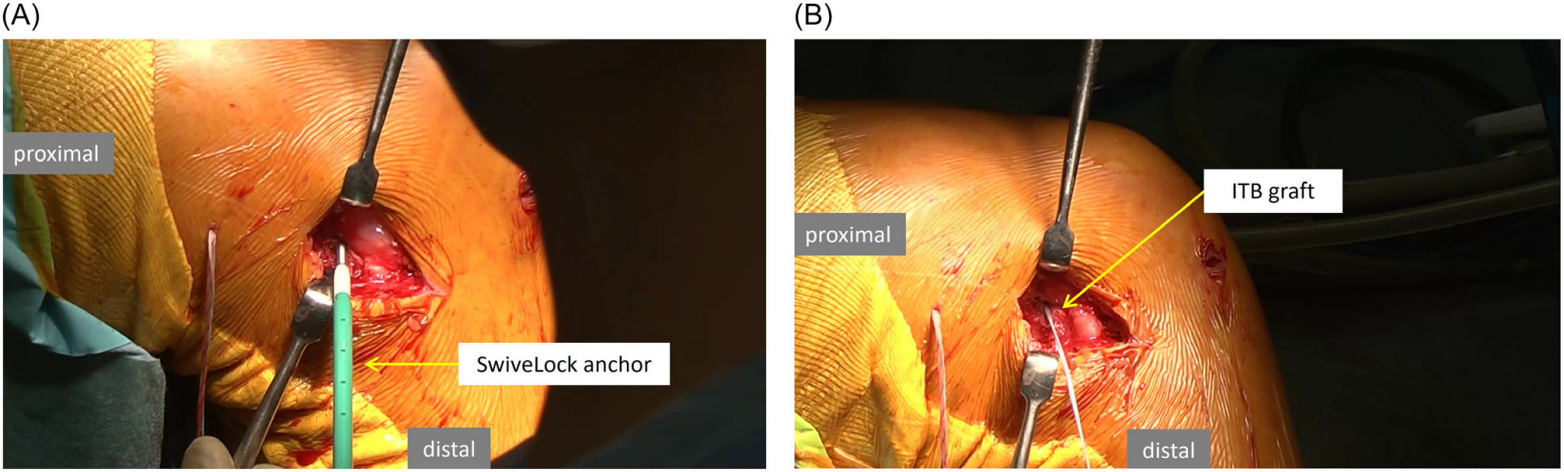
Intraoperative image with the patient supine and the right knee flexed to approximately 90°. (A) While maintaining the appropriate tunnel trajectory, a 4.75‐mm SwiveLock anchor (Arthrex, Naples, FL) is inserted along the graft. With the knee in slight flexion and the lower leg held in internal rotation, maximal tension is applied to the SutureTape, and fixation is achieved. (B) After anchor insertion, the sutures are removed, completing LET fixation. (ITB, iliotibial band; LET, lateral extra‐articular tenodesis.)

**FIGURE 16:**
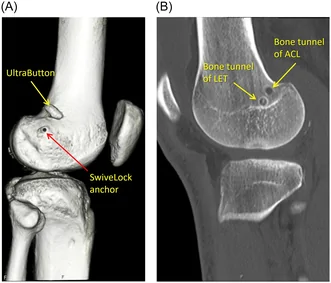
Postoperative CT images of the right knee. (A) Three‐dimensional CT reconstruction showing the LET fixation screw positioned approximately 5 mm proximal and posterior to the lateral femoral epicondyle. The ACL femoral fixation button is located proximal to the screw, with no tunnel convergence observed. (B) Sagittal CT image confirming no significant convergence between the ACL and LET femoral tunnels. Minor contact between tunnels is considered acceptable when drilling is performed in this sequence. (ACL, anterior cruciate ligament; CT, computed tomography; LET, lateral extra‐articular tenodesis.)

**TABLE 3:** Postoperative Rehabilitation Protocol

**Timepoint**	**Protocol**
Immediately postoperative	Knee immobilized in splint; nonweight‐bearing
Postoperative day 4	Progression to full weight‐bearing with ACL brace
3 mo	Jogging permitted; ACL brace discontinued
6 mo	Return to sports permitted (contingent on muscle strength recovery)
Meniscal repair cases	Weight‐bearing progression adjusted according to repair technique

ACL, anterior cruciate ligament; mo, months.

**TABLE 4:** Advantages and Disadvantages

**Advantages**	**Disadvantage**
• Simple modification of the surgical sequence without requiring specialized instruments	• Reliance on intraoperative fluoroscopy
• Real‐time fluoroscopic assessment allows immediate trajectory adjustment during ACL tunnel creation	• Requires familiarity with fluoroscopic imaging in the figure‐4 position
• Reduces the risk of ACL graft damage caused by femoral tunnel convergence	• Creating the ACL femoral tunnel with the passing pin left in place adds procedural complexity
• May decrease operative time by avoiding tunnel recreation	• Minor tunnel contact cannot be completely eliminated
• Applicable to other lateral extra‐articular procedures, including ALL reconstruction	

ACL, anterior cruciate ligament; ALL, anterolateral ligament.

**TABLE 5:** Pearls and Pitfalls

**Pearls**	**Pitfalls**
• Identify the femoral attachment of the LCL precisely before drilling	• Incorrect LCL landmark identification may result in malpositioned LET tunnel
• Drill the LET tunnel prior to ACL femoral tunnel to minimize convergence risk	• Poor fluoroscopic positioning can lead to inaccurate tunnel trajectory
• Maintain fluoroscopic true lateral view during guide pin placement	• The transportal technique may not allow optimal tunnel placement; prepare for outside‐in as needed
• Leave LET guide pin in place during ACL drilling for real‐time convergence assessment	• Excessive graft tension during LET fixation may lead to overconstraint of the lateral compartment
• If potential tunnel convergence is anticipated, direct the ACL guide pin more proximally	

ACL, anterior cruciate ligament; LCL, lateral collateral ligament; LET, lateral extra‐articular tenodesis.

## DISCUSSION

This technique modifies the operative sequence when combining ACL reconstruction with LET. Specifically, the LET femoral tunnel is created before the ACL femoral tunnel to reduce graft damage caused by femoral tunnel interference. This concept is applicable not only to LET but also to ALL reconstruction, where tunnel convergence may similarly compromise the ACL graft.

The modified Lemaire technique allows the ITB to pass posterior to the LCL, creating a pulley effect that controls rotational instability. Biomechanical studies have shown that the addition of LET significantly reduces intra‐articular ACL graft forces during anteriorly directed loading.^29^

The femoral fixation point in the modified Lemaire technique has not been uniformly defined.^16^, ^28^, ^33^, ^34^, ^35^ At our institution, the LET femoral tunnel is created just proximal and posterior to the femoral attachment of the LCL, which corresponds to approximately 5 mm proximal and posterior to the lateral femoral epicondyle and closely approximates the classic Lemaire point.^9^ Although the ACL femoral tunnel exit point can be controlled under fluoroscopy to be located more proximally, the LET femoral fixation point is relatively constrained. Therefore, creating the more constrained LET femoral tunnel first represents a rational strategy.

A key aspect is maintaining the LET passing pin in place during ACL femoral tunnel creation, allowing real‐time fluoroscopic assessment and immediate trajectory adjustment if contact is detected. Wasdev et al.^36^ reported that eccentric superior deviation of the ACL femoral tunnel is acceptable when soft‐tissue grafts and suspensory fixation are used, whereas central violation requires redirection and recreation of the LET femoral tunnel.

Recent studies have reported tunnel convergence rates of 47% to 80% depending on knee flexion angle during ACL tunnel drilling.^27^, ^28^, ^30^ Although tunnel convergence may become less problematic with experience, the high incidence underscores its clinical relevance.^2^, ^3^ The present technique reduces graft damage risk and may decrease operative time by avoiding tunnel recreation.

Alternative strategies include creating the ACL femoral tunnel first with arthroscopic inspection and tunnel recreation if interference occurs;^26^ however, this may necessitate repeated drilling. Knotless anchor fixation reduces convergence risk because of small drill diameters (1.8‐2.6 mm),^24^, ^25^, ^37^, ^38^ although graft damage risk cannot be entirely eliminated.

A limitation is the reliance on fluoroscopy. At our institution, fluoroscopy is routinely employed even in standard ACL reconstruction. However, concerns regarding radiation exposure may limit widespread adoption. With sufficient experience, the procedure can be performed without fluoroscopy.

In conclusion, creating the LET femoral tunnel prior to the ACL femoral tunnel is a simple modification that minimizes femoral tunnel interference and subsequent ACL graft damage. This technique is applicable to LET using the modified Lemaire technique and may be extended to other LEAP, including ALL reconstruction. Requiring no specialized instruments, this approach provides a safe option for combined ACL reconstruction with LEAP in patients at high risk of graft failure.

## DECLARATION OF GENERATIVE AI AND AI‐ASSISTED TECHNOLOGIES IN THE WRITING PROCESS

During the preparation of this work, the authors used ChatGPT (OpenAI) to assist with English translation and language refinement. The authors reviewed and edited all content and take full responsibility for the accuracy and integrity of the manuscript.

## DISCLOSURES

The authors (M.S., T.K., Y.Y., H.L., K.N.) declare that they have no known competing financial interests or personal relationships that could have appeared to influence the work reported in this article.

## ACKNOWLEDGMENTS

The authors thank Mr. Gary Cooper for his contribution to the narration of the surgical video.
